# Evaluation of the Efficacy of a Full-Spectrum Low-THC *Cannabis* Plant Extract Using In Vitro Models of Inflammation and Excitotoxicity

**DOI:** 10.3390/biom14111434

**Published:** 2024-11-11

**Authors:** Emily Ross-Munro, Esra Isikgel, Bobbi Fleiss

**Affiliations:** 1 School of Health & Biomedical Sciences, RMIT University, Bundoora, VIC 3083, Australia; emily.ross-munro@rmit.edu.au; 2 Fenix Innovation Group Pty Ltd., Melbourne, VIC 3149, Australia; esra@fenixinnovationgroup.com

**Keywords:** autism, botanical synergy, neuroinflammation, full spectrum, neurodevelopmental disorders

## Abstract

Evidence has accumulated that *Cannabis*-derived compounds have the potential to treat neuroinflammatory changes present in neurodevelopmental conditions such as autism spectrum disorder. However, research is needed on the specific brain health benefits of strains of whole *Cannabis* extract that are ready for commercial production. Here, we explore the anti-inflammatory and neuroprotective effects of NTI-164, a genetically unique high-cannabidiol (CBD), low-Δ9-tetrahydrocannabinol extract, and also CBD alone on BV-2 microglia and SHSY-5Y neurons. Inflammation-induced up-regulation of microglial inflammatory markers was significantly attenuated by NTI-164, but not by CBD. NTI-164 promoted undifferentiated neuron proliferation and differentiated neuron survival under excitotoxic conditions. These effects suggest the potential for NTI-164 as a treatment for neuropathologies.

## 1. Introduction

Despite differences in clinical profiles, genetics, and symptoms between neurodevelopmental disorders, many share immune dysregulation as an underlying pathophysiological mechanism [1,2,3,4,5]. In particular, the role of the neuroimmune axis in the pathogenesis of the neurodevelopmental disorder autism spectrum disorder (ASD) has been the focus of intense research during the past decade [4,6,7,8,9]. Despite the rising prevalence of neurodevelopmental disorders [10,11] and their significant economic burden [12,13], we have limited effective pharmacological therapies [14,15].

Evidence highlights the role of glial cells and, specifically, the disruption of homeostatic microglial function as directly contributing to the etiopathogenesis of many neurodevelopmental disorders [4,16,17,18,19]. Microglia are myeloid cells of mesodermal origin that mediate key developmental processes, including neuronal migration, synaptogenesis, oligodendrocyte maturation, and synaptic remodelling (pruning), as well as a diverse array of physiological functions in adulthood, such as the regulation of adult neurogenesis [6,20,21,22]. In response to signs of homeostatic disturbance, microglia shift activity states and transform from homeostatic to an ‘immune-activated state’ [23]. Depending on the pathological condition and its impacts on the local microenvironment, microglia contribute various immune-related activities through the differential release of molecules, including pro-inflammatory and anti-inflammatory cytokines and neurotrophins [24,25,26]. Historically, we have divided microglia’s activation states in response to brain insults into two reactive phenotypes: an M1 pro-inflammatory response and an M2 neuroprotective response [21,24]. Although this is an oversimplification and these cells exist across a continuum in vivo [27], using a simplified nomenclature based on a well-established set of immune-activated-state markers is helpful for basic function descriptions in simplified systems, such as in vitro paradigms like those in this study.

The endocannabinoid system (ECS) is composed of the endogenous cannabinoids, their receptors (CB1 and CB2) and associated metabolic enzymes. The ECS is a critical regulator of early neuronal plasticity [28] and behavioural processes [29]. In immune-activated microglia, CB2 receptors modulate synaptic activity and neuroinflammatory responses [30]. A large body of evidence further supports the involvement of ECS in ASD-related neurodevelopmental processes since aberrant endocannabinoid signalling pathways are linked to ASD pathogenesis [31,32,33]. Furthermore, recent clinical studies in children with ASD have shown altered endocannabinoid-CB2 cellular signalling [34] and circulating endogenous cannabinoids [35,36], underpinning the importance of ECS as a potential therapeutic target for ASD and related neuroinflammatory diseases.

The growing evidence for the participation of ECS in neurodevelopmental processes, in parallel with the well-researched therapeutic potential of medicinal *Cannabis*, has raised interest in the use of *Cannabis*-derived compounds to treat neurodevelopmental disorders. For centuries, the therapeutic benefits of *Cannabis sativa* L. extracts have been widely recognised in the treatment of a broad spectrum of nervous-system-related conditions ranging from common neurological and hyperexcitability disorders such as epilepsy and affective disorders (e.g., anxiety) to neurodegenerative disorders such as Alzheimer’s disease [37]. The role of the most abundant nonpsychoactive phytocannabinoid cannabidiol (CBD) in the modulation of inflammatory and immune responses has been extensively documented [38,39,40]. The most current research on the treatment of common neurological and psychiatric disorders is focused on the application of CBD alone or in combination with the main psychoactive *Cannabis* component Δ9-tetrahydrocannabinol (THC) [41,42,43,44]. Evidence from experimental models suggests that individual phytocannabinoids derived from the *Cannabis sativa* plant modulate microglial functions by blocking excitotoxic-induced activation [45], inhibiting intracellular calcium increase [46] and modulating inflammatory responses [47,48]. 

Clinical evidence suggests that to improve the safety profile and improve the therapeutic effects of *Cannabis*-based treatments, we should administer CBD combined with other phytocannabinoids [35,49,50,51]. Nearly 25 years ago, the term ‘entourage effect’ was coined to describe the synergistic contributions of many endogenous cannabinoids in *Cannabis* [52]. The benefits of the entourage effect over single isolates have been demonstrated in preclinical models of neurological disorders [53,54] and cancer [55,56,57]. Increasingly, specific breeding programs are generating *Cannabis* lines with low THC, often known as hemp plants, but with higher levels of other neuroactive compounds [58]. Also, the use of cannabinoid extracts is possible in people with neurodevelopmental disorders, as although cannabinoids have a low uptake into the brain after oral delivery, they have high chemical potency. This potency is illustrated well by the example of THC, where only 6% is bioavailable when ingested orally. Of that, only 1% crosses the blood–brain barrier, but the effects on neurological function are substantial [59]. Similar potency data are available for CBD, and although less is known about the minor cannabinoids, their efficacy when applied orally in preclinical models of central nervous system (CNS) disorders is notable [37,40,60]. Also, altered blood–brain permeability is commonly reported in studies of people with neurodevelopmental disorders and preclinical models [61], including ASD [62,63], further suggesting a valuable role of oral treatment in these disorders.

Therefore, this study’s primary focus was to investigate the effect of a full-spectrum medicinal hemp strain *Cannabis* plant extract with only 0.08% THC (NTI-164) on inflammatory and excitotoxic responses in well-established preclinical in vitro models.

## 2. Materials and Methods

Unless otherwise stated, all reagents were sourced from Sigma-Aldrich (St. Louis, MO, USA).

### 2.1. Isolation of NTI-164 Extract

The NTI-164 *Cannabis sativa* plant was grown and harvested following the natural open pollination process. After harvesting, we ground the dried plant using a commercial herb grinder until the particulates were approximately 1 mm in diameter. The ground plant was then mixed in a ratio of 1 g to 10 mL of 100% ethanol, and then, this mixture was placed on a rocker at 50 rpm for 4 h. We then aspirated the mixture into a new tube, and the remaining particulates were removed by centrifugation at 300× *g* for 10 min. We stored the final extract at −20 °C until use, and an aliquot was sent for analysis with ultra-high-performance liquid chromatography (U-HPLC) to assess the purity and composition of the final extract or for use in cell culture work.

### 2.2. Ultra-High-Performance Liquid Chromatography (U-HPLC)

We performed the experiments using a Waters H Class UPLC system coupled to a photodiode array detector and quadruple Dalton mass detector—a single-quadrupole mass spectrometer (MS) detector with an electrospray ionisation (ESI) interface. Separation was performed using Waters Cortecs UPLC Shield C_18_ 1.6 µm, 2.1 × 100 mm column. The Mobile Phase was an isocratic run of aqueous 0.1% trifluoracetic acid buffer and acetonitrile. The sample (0.1 g) was weighed and dissolved in tetrahydrofuran and diluted with acetonitrile (10 mL) before we performed a second dilution before analysis. Relative Response Factors (RRFs) using a certified CBD standard were employed for the quantitation of all cannabinoids. We achieved this by generating a 6-point calibration curve of each cannabinoid in two separate cannabinoid mixtures consisting of a Major and Minor cannabinoid mix. The Major mixture consisted of cannabidiol (CBD), cannabidiolic acid (CBDA), Δ9-tetrahydrocannabinol (Δ9-THC) and Δ9-tetrahydrocannabinolic acid A (Δ9-THCA). The Minor mixture included cannabidivarin (CBDV), cannabigerol (CBG), cannabinol (CBN), tetrahydrocannabivarin (THCV), tetrahydrocannabivarinic acid (THCVA), cannabigerolic acid (CBGA), Δ8-tetrahydrocannabinol (Δ8-THC), cannabichrome (CBC), cannabicyclol (CBL) and cannabinolic acid (CBNA). The RRF was calculated using the formula below.

$$\mathrm{RRF}=\frac{\mathrm{Slope}\;\mathrm{of}\;\mathrm{Reference}\;\mathrm{Cannabinoid}\;\left(\mathrm{CBD}\right)}{\mathrm{Slope}\;\mathrm{of}\;\left(\mathrm{Major}\;\mathrm{or}\;\mathrm{Minor}\right)\;\mathrm{Cannabinoid}}$$

Certified Reference standard solutions (1 mg/mL) were obtained from Cerilliant Corporation (Round Rock, TX, USA), using the local Australian supplier Novachem, Pvt Ltd. (Heidelberg West, Victoria, Australia)

### 2.3. Microglial BV2 Cell Culture

The immortalised microglia cell line, BV2 (EP-CL-0493, Elabscience, Houston, TX, USA), was cultured in Rosewell Park Memorial Institute (RPMI) 1640 medium containing 2 mM L-glutamine (Gibco, Cat# 11875, Waltham, MA, USA), containing 0.1% gentamycin (Thermo, Waltham, MA USA. Cat# 15750060) and supplemented with heat-inactivated newborn calf serum (NCS; Gibco, Cat# 26010-074, Waltham, MA, USA) at a concentration of 10% for expansion and 5% when plated for experiments. All cells were from between passage numbers 5 and 10. BV2 were plated at 45,000 cells/cm^2^ and treated 24 h after plating. Cells were treated with phosphate-buffered saline (PBS; Gibco, Cat# 100100, Waltham, MA, USA) to serve as a control (i.e., unstimulated cells) or interleukin-1β (IL-1β; 50 ng/mL; Miltenyi Biotec, Cat#130-101-682, Bergisch Gladbach, NRW, Germany) plus interferon-γ (IFNγ; 20 ng/mL; Miltenyi Biotec, Cat# 130-105-785, Bergisch Gladbach, NRW, Germany) to induce inflammation (i.e., immune activation). We based the cell culturing and immune activation techniques described here on established protocols from our lab [64,65,66]. After one hour of treatment with either PBS or IL-1β+ IFNγ, we applied a 10 µL dose of NTI-164 extract (1/3000 dilution of EtOH extract into PBS based on pilot experiments) or CBD (1 mg/mL, based on a detailed literature search [67,68,69,70]), and we performed the analysis 24 h after treatment. Treatment times were based on our previous experiments, and we designed the experiment to focus on observing the direct effects of treatments using a 24 h timepoint. In contrast, longer incubations lead to autocrine signalling by the microglia [24]. As NTI-164 extract and CBD were dissolved in ethanol for this investigation, all control wells (PBS-treated) also contained a matched ethanol concentration. An independent replicate (*n* = 1) was cells plated and treated from one stock flask per day. The dose of CBD used to treat the BV2 (and SHSY-5Y, below) was 10 μM, chosen from a literature review based on the most effective and non-toxic dose [67,68,69,71]. Assays were terminated 24 h after treatment to allow for the direct and specific effects of the agents (CBD, NTI164, details below) to be observed rather than autocrine signalling from the ongoing conditioning of the media by the cells.

### 2.4. Neuronal SHSY-5Y Cell Culture

The immortalised neural precursor cell line, SHSY-5Y (The American Type Culture Collection, Cat# CRL-2266, Manassas, VA, USA), was used to assess the effect of NTI-164 or CBD treatment on neuronal differentiation or in response to excitotoxic injury. For expansion, we cultured cells in Dulbecco’s modified eagle medium (DMEM, Gibco, Cat# 11995, Waltham, MA, USA) that contained 1 g/L of D-glucose, 584 mg/L of L-glutamine, 110 mg/L sodium pyruvate, and phenol red, further supplemented with 0.1% penicillin-streptomycin (Sigma-Aldrich, Cat# P4458) and 10% NCS. We plated SHSY-5Y cells at 45,000 cells/cm^2^ for experiments. For differentiated cell work, we altered the medium to contain 1% NCS and daily delivery of 10 µM all-trans-retinoic acid (Sigma-Aldrich, Cat# R2625 dissolved in PBS). We differentiated the cells for five days with daily changes in the half-volume media before excitotoxicity assays. We exposed SHSY-5Y to 3 mM glutamate (Sigma-Aldrich, Cat# G815) dissolved in PBS, administered as a 10 µL dose. After one hour, cells were treated with NTI-164 or CBD as previously described for BV2 experiments, and analysis was carried out 24 h after treatment. An independent replicate (*n* = 1) was cells plated and treated from one stock flask per day.

### 2.5. Multiplex Cytokine/Chemokine Assay

Sterile 96-well plates were incubated for 30 min at room temperature with 300 µL of 10% bovine serum albumin (BSA) dissolved in PBS (BSA buffer). We then discarded the BSA solution, air-dried the plates and used these protein-blocked plates to collect BV2 culture medium 24 h after initiation of the treatment protocol. The medium was immediately centrifuged to remove particulate at 300× *g* for 10 min with supernatant, then transferred to an additional BSA-blocked plate and stored at −80 °C until use in multiplex experiments, as per our previous work [72,73]. We measured the levels of cytokines and chemokines in the medium using a Bio-Plex 200 according to the manufacturer’s instructions (Bio-Rad, Cat# M60000007A, Hercules, CA, USA). As per our previous work, the measured cytokines and chemokines included IL-2, IL-10, IL-5, GM-CSF, and TNFα [74,75]. We ran all samples in duplicate with data analysed via Bio-Plex Manager software (Version 5.7) from BioRad.

### 2.6. Immunohistochemistry

According to previous work from the lab [76,77], plated cells were fixed for 10 min with 2% paraformaldehyde (PFA; VWR Chemicals, Cat# 28794.364, Radnor, PA, USA) in phosphate buffer and washed for 3 × 5 min with PBS. All washes were for 3 × 5 min throughout the protocol. The plates were stored at 4 °C in 150 µL of PBS containing 0.02% sodium azide (Sigma-Aldrich, Cat# S-2002). Immediately before staining, cells were washed and then incubated for 30 min with 100 µL of BSA buffer containing 0.01% Triton X (Sigma-Aldrich, Cat# X100). Subsequently, we removed 75 µL of BSA buffer and added 25 µL of primary antibody diluted in PBS to obtain the final concentration (Table 1). We incubated the plates overnight at 4 °C and washed them the following day. Subsequently, 50 µL of the corresponding fluorescent secondary antibody (Table 1) was applied for 1 h at room temperature, removed and the well was washed. To visualise cell nuclei, 4′,6-diamiino-2-phenylindole (DAPI; Invitrogen, Cat# D21490, Waltham, MA, USA) diluted 1:1000 in PBS was applied for 15 min at room temperature with plates subsequently washed. For imaging, photomicrographs were taken using the EVOS M5000 (Invitrogen, Cat# AMF5000) in three fields of view per well from duplicate wells and analysed using Fiji 2 (Version 2.9.0/1.53t) to determine the area coverage of each marker. We conducted the staining and analysis as per our previous work [24,78]. Specifically, the percentage area coverage was determined by calculating the number of pixels in an image of positive staining divided by the total pixel number and then multiplied by 100. Area coverage was determined in fixed-size images, from three images per well, and two wells were stained per replicate. Area coverage of DAPI was used as a surrogate for cell number, and we adjusted outputs for inflammatory markers to DAPI to account for variance in cell number per image.

### 2.7. Cell Viability Assay (Mitochondrial Respiration Activity)

We evaluated mitochondrial respiratory activity as per previous work [64,79], using the 3-(4,5-dimethylthiazol-2-yl-)-2,5-diphenyl-2H-tetrazolium bromide (MTT) assay (Sigma-Aldrich, Cat# M6494). We added MTT to the wells at a final concentration of 250 µg/mL. After 30 min, we carefully removed and discarded the medium, then dissolved the formazan within the wells in dimethylsulfoxide (DMSO; Sigma-Aldrich, Cat# D2650, St. Louis, MO, USA), and the absorbance was measured at 490 nm using a spectrophotometer (Glomax Multi+; Promega, Chilworth Guildford, UK). Previous studies in our lab have shown that with this dose of MTT, cell density and incubation time, this time of analysis (30 min) is within the linear range for increases in optical density in the assay.

### 2.8. Statistics

We averaged the data for replicates within experiments, and then, data from at least three independent experiments were analysed using GraphPad Prism software (V10, GraphPad Software, Inc., San Diego, CA, USA). We have outlined the replicate numbers for each experiment in the figure legends. A one-way ANOVA or Kruskal–Wallis test was used to compare the impact of treatments on cell responses, with Šídák’s post hoc test to interrogate the specific group differences using the control group (excipient-only) or the inflammation-only group as the comparison. We specifically compared NTI164 vs. control or inflammation, CBD vs. control or inflammation and NTI-164 vs. CBD. We expressed all data as mean ± standard error of the mean (SEM) with significant differences between groups set at a *p*-value of less than 0.05.

## 3. Results

### 3.1. Chemical Characterisation of NTI-164 Components by U-HPLC

**Figure 1:**
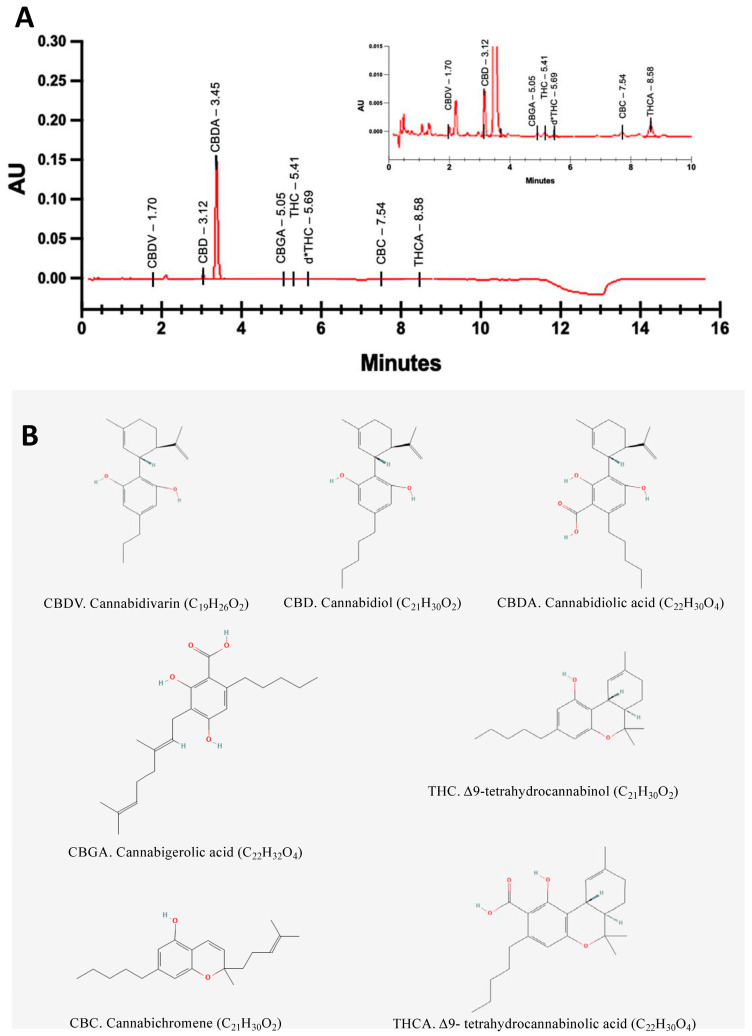
(**A**) A representative HPLC chromatogram of the NTI-164 extract. (**B**) Two-dimensional molecular structure and molecular formula for the indicated cannabinoids (from PubChem). Abbreviations. AU: Absorbance units; CBDV: Cannabidivarin; CBD: cannabidiol; CBDA: cannabidiolic acid; CBGA: Cannabigerolic acid; CBC: Cannabichromene; THCA: Δ9-tetrahydrocannabinolic acid; THC: Δ9-tetrahydrocannabinol.

U-HPLC analysis revealed that NTI-164 was rich in the acidic precursor of CBD, CBDA (1.110 mg/mL), and the extract also contained CBD (0.165 mg/mL), CBDV (0.023 mg/mL), CBGA (0.050 mg/mL), CBC (0.023 mg/mL), THC (0.025 mg/mL), d*-THC (0.013 mg/mL), and THCA (0.048 mg/mL). The HPLC chromatogram was recorded at 229 nm, and we show an example in Figure 1 together with the 2D structures of the compounds analysed.

### 3.2. Effect of NTI-164 on Mitochondrial Function and Number of BV-2 Microglia

**Figure 2:**
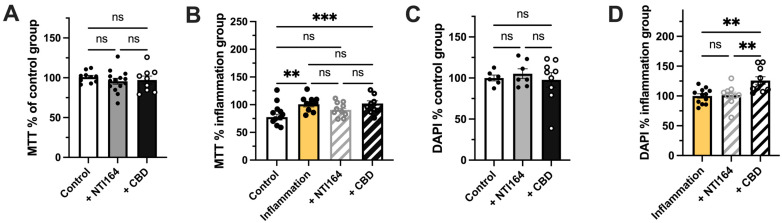
Effect of NTI-164 and CBD on MTT outputs in BV-2 microglia exposed only to excipients (control, white), excipient + NTI164 (+NTI164, grey), excipient + CBD (+CBD, black) excipient + IL-1β and IFNγ (inflammation, yellow) and inflammation + NTI164 (+NTI164, grey hatched) and inflammation + CBD (+CBD, black hatched) in (**A**) unstimulated condition, (**B**) immune-activated condition and overall DAPI-positive cell number in (**C**) unstimulated condition, (**D**) immune-activated condition. Mean ± SEM, *n* = 6–10. One-way ANOVA, with post hoc test as indicated on the graphs. Significance set at **, *p* < 0.01; ***, *p* < 0.001. ns = *p* > 0.05.

In unstimulated BV2 microglial cells (treated with PBS + EtOH excipient), NTI-164 or CBD treatment had no significant effect on mitochondrial activity as assessed via the MTT assay (Figure 2A). In line with prior findings, we observed that mitochondrial activity was significantly increased in immune-activated cells (stimulated by IL-1β and IFNγ + EtOH excipient) versus control (Figure 2B). While NTI-164 prevented this increase (Control vs. +NTI164, ns), CBD did not (Figure 2B, Control versus +CBD, *p* < 0.01). In line with this, exposure to NTI-164 or CBD in unstimulated cells did not significantly alter overall cell number as assessed via area-coverage analysis of DAPI staining (Figure 2C). Interestingly, CBD significantly increased the area coverage of DAPI staining in immune-activated cells. As such, CBD significantly differed from the NTI-164 response as NTI-164 did not alter the DAPI staining from the immune-activated cells (Figure 2D).

### 3.3. Effect of NTI-164 on the Expression of Inflammatory Markers in BV-2 Microglia

**Figure 3:**
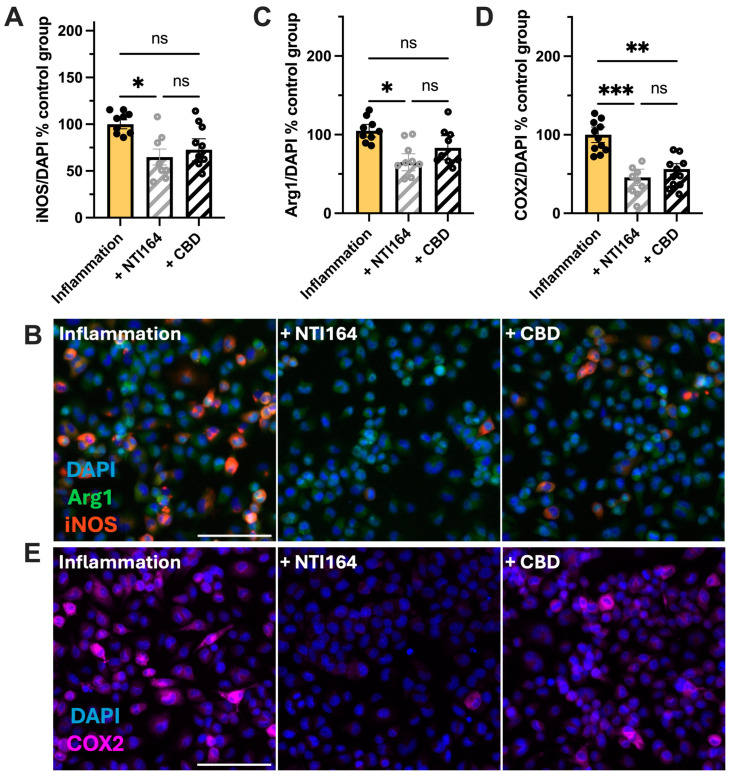
Effect of NTI-164 and CBD on the expression of inflammatory markers in BV-2 microglia cells exposed to excipient + IL-1β and IFNγ (inflammation, yellow) and inflammation + NTI164 (+NTI164, grey hatched) and inflammation + CBD (+CBD, black hatched) in (**A**,**B**) inducible-nitric oxide synthase (iNOS), (**C**) arginase-1 (Arg1) and (**D**,**E**) expression of cyclo-oxygenase-2 (COX2) protein normalised to DAPI-positive cell number in the control group (PBS only). Scale bar = 100 µM. Mean ± SEM, *n* = 8–9. One-way ANOVA, with post hoc test as indicated on the graphs. Significance set at *, *p* < 0.05; **, *p* < 0.01; ***, *p* < 0.001. ns = *p* > 0.05.

As expected, immune-activated microglia expressed more nitric oxide synthase (iNOS), arginase 1 (Arg1) and cyclooxygenase 2 (COX2) than controls (Supplementary Figure S1A) [24,64]. Treatment of immune-activated microglia with NTI-164 resulted in a significant decrease in iNOS and Arg1 expression, which was not observed in cells treated with CBD (Figure 3A,B). NTI-164 and CBD significantly reduced the expression of COX-2 from immune-activated microglia (Figure 3C).

### 3.4. Effects of NTI-164 on Microglial Cytokine Production

**Figure 4:**
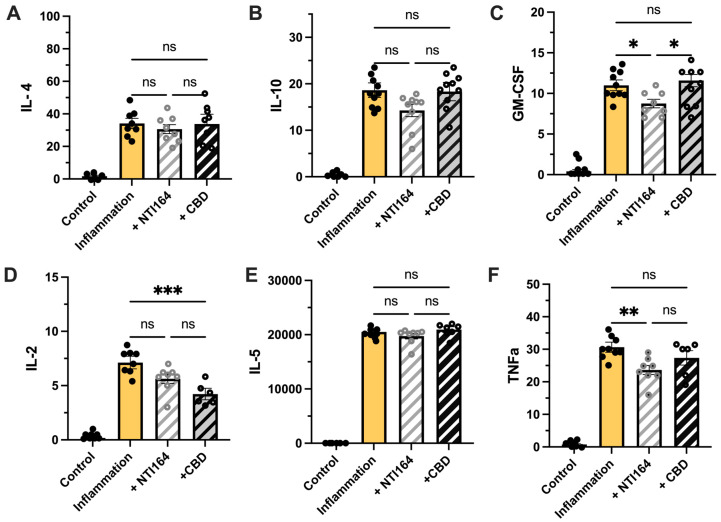
Impact of NTI-164 and CBD on inflammation-induced cytokine release from BV2 microglia exposed to only excipient (control, white), excipient + IL-1β and IFNγ (inflammation, yellow) and inflammation + NTI164 (+NTI164, grey hatched) and inflammation + CBD (+CBD, black hatched). In (**A**) interleukin-4 (IL-4), (**B**) interleukin-10 (IL-10), (**C**) granulocyte-macrophage colony-stimulating factor (GM-CSF), (**D**) interleukin-2 (IL-2), (**E**) interleukin-5 (IL-5) and (**F**) tumour necrosis factor-alpha (TNF-α) analysed by multiplex immunoassay and expressed as fluorescent units. Mean ± SEM, *n* = 8–9. One-way ANOVA, with post hoc test as indicated on the graph. Significance set at *, *p* < 0.05; **, *p* < 0.01; ***, *p* < 0.001. ns = *p* > 0.05.

As expected, immune-activated microglia expressed more cytokines [64]. In immune-activated BV2 cells, treatment with NTI-164 or CBD did not cause significant changes in the levels of the anti-inflammatory cytokines IL-4 and IL-10 (Figure 4A,B). However, treatment with NTI-164 significantly reduced the growth and differentiation-inducing chemokine GM-CSF expression, but this finding was not observed in cells treated with CBD (Figure 4C). Thus, there was a significant difference between NTI-164 and CBD in levels of GM-CSF (Figure 4C). CBD significantly decreased pro-inflammatory IL-2 production, but NTI-164 did not (Figure 4D). There was no effect on IL-5 of NTI-164 or CBD. Finally, NTI-164, but not CBD, significantly reduced levels of TNFα (Figure 4F).

### 3.5. Effect of NTI-164 and CBD on SHSY-5Y Neurons

**Figure 5:**
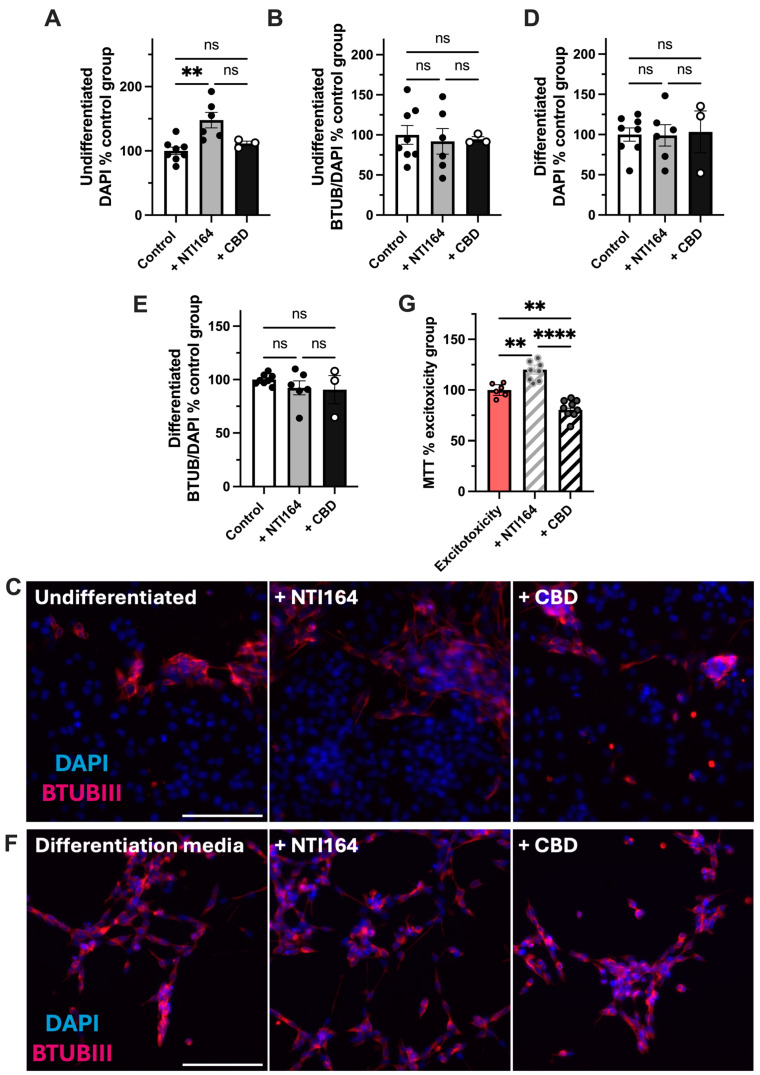
Impacts of NTI-164 and CBD on SHSY-5Y neurons exposed only to excipients (control, white), excipient + NTI164 (+NTI164, grey), excipient + CBD (+CBD, black) excipient + excitotoxicity (Excitotoxicity, pink) and excitotoxicity + NTI164 (+NTI164, grey hatched) and excitotoxicity + CBD (+CBD, black hatched). (**A**) Impacts on the number of DAPI-positive cells in undifferentiated cultures; (**B**) impacts on the expression of beta-tubulin (BTUB) as a proportion of DAPI-positive cells as an indicator of spontaneous differentiation, with representative images in (**C**). (**D**) Impacts on the number of DAPI-positive cells in differentiated cultures. (**E**) Impacts on the expression of BTUB in differentiated cells, with representative images in (**F**). (**G**) The impacts on the MTT output (mitochondrial activity assay) in differentiated cells in the glutamate exposure excitotoxicity assay. Scale bar = 150 µM, same across all panels. Mean ± SEM, *n* = 3–10. One-way ANOVA with post hoc test as indicated on the graph. Significance set at **, *p* < 0.01; ****, *p* < 0.0001. ns = *p* > 0.05.

In undifferentiated neurons, NTI-164 treatment significantly increased the area coverage of DAPI staining, while CBD treatment did not result in a detectable change in DAPI staining (Figure 5A). However, neither treatment induced spontaneous differentiation as assessed by expression of Beta III tubulin (Figure 5B). In differentiated neurons, neither NTI-164 nor CBD altered the number of cells or the amount of differentiation (Figure 5C,D). In a paradigm of excitotoxicity leading to approximately 20% reduction in MTT outputs (Supplementary Figure S1B,C), NTI-164 significantly increased the mitochondrial activity (survival) of neurons. CBD did not rescue mitochondrial activity, and there was a significant difference between the MTT in CBD-treated and NTI-164-treated (Figure 5G).

## 4. Discussion

The current study sought to determine the neuroprotective potential of a full-spectrum medicinal *Cannabis* plant extract (NTI-164) using in vitro models of neuroinflammation and neuroinjury. In our study, NTI-164 normalised inflammation-induced changes in immune-activated microglia cells by preventing increased mitochondrial activity, decreasing the expression of pro-inflammation-associated markers (COX2, iNOS, GM-CSF, and TNFa) and promoting the survival of differentiated neurons under excitotoxic conditions. As a common feature of neurodegenerative and neurodevelopmental disorders, neuroinflammation typically involves the activation of resident immune cells in the CNS (glia cells) and production of cytokines together with reactive oxygen intermediates that result in infiltration of immune cells, damage to the integrity of the blood–brain barrier and excitotoxicity [80].

Phytocannabinoids or exogenous cannabinoids are abundant in the *Cannabis sativa* plant and have been found to influence the inflammatory response through various biological mechanisms, including the regulation of microglia [45,46]. The therapeutic benefits of combined phytocannabinoids have been consistently demonstrated in several experimental models [46,68,81,82,83] and clinical research studies [84], in particular, for the treatment of ASD-related symptoms [35,50,85,86,87]. In an animal model of amyotrophic lateral sclerosis, daily treatment of mice with a phytocannabinoid-enriched botanical extract (Sativex) for 20 weeks resulted in a significant decrease in the progression of neurological impairment [83]. In a multiple sclerosis model, Sativex treatment for ten days reduced microglial immune-related activity and the expression of pro-inflammatory cytokines [82]. In a more recent experimental study, combination treatment with THC and CBD (but not either compound alone) in a murine multiple sclerosis model led to reduced levels of pro-inflammatory cytokines such as IL-6 and TNF- while increasing anti-inflammatory cytokine production of anti-inflammatory cytokines [88]. The authors proposed the underlying anti-inflammatory and neuroprotective mechanism of combined phytocannabinoids involves decreased cell cycle arrest and apoptosis, regulated by miRNA-mediated signalling pathways [88].

In this study, the full-spectrum *Cannabis* extract NTI-164 effectively attenuated elevated inflammation-induced mitochondrial activity. The links between mitochondrial function and inflammatory responses are complex [77,89]. MTT outputs are also a snapshot of the total cell NAD/NADH ratio, not only mitochondrial function, as NADH is primarily responsible for reducing MTT to formazan [90]. Immune activation leads to a switch to increased glycolysis (more NADH/less ATP) and increased mitochondrial reactive oxygen species production [77,89]. However, maintaining higher levels of oxidative phosphorylation drives a stronger pro-inflammatory state [91], showing that the switch is not obligatory for an immune reaction to occur. Confounding the interpretation of any MTT data is also the interaction between changes in proliferation and cell survival, as overall changes in cell number and changes in the NADH reserve of each cell impact the MTT output. These variables coalesce, such that early after exposure to inflammation, BV2 microglia have previously been shown to increase MTT output (in agreement with our findings) but decrease MTT at later timepoints [92]. Thus, our data showing that NTI-174 made the MTT data more like control outputs and less like inflammatory outputs may reflect a normalisation of an early glycolytic burst, as reported by others [93].

We study microglia as they are responsible for immune surveillance [94] and responses to almost all kinds of injury and neurological disorder [18,25,26]. Of particular interest is that in children with ASD, microglia activation and expression of inflammatory genes are altered in multiple brain regions, providing strong evidence that neuroinflammation is involved in ASD pathogenesis [95,96,97]. *Cannabis* extracts may offer potential therapeutic benefits by targeting microglia-induced neuroinflammation in neuropathology. One of the chief mechanisms by which microglia orchestrate neuroinflammation is the production of cytokines and bioactive lipids [76,98]. Both NTI-164 and CBD significantly decreased COX-2 levels in immune-activated BV2 microglia. Previous studies have shown that whole *Cannabis* extract [99] and the acidic cannabidiol CBDA [100] exhibit anti-inflammatory effects by inhibiting COX-2 activity in cell culture. NTI-164 but not CBD significantly decreased the inflammation-induced expression of iNOS, GM-CSF and TNFa, inflammatory markers whose increased expression is associated with responses to brain injury and neurodegenerative disorders [73,75]. CBD has previously been reported, at the same dose used in our study, to effectively decrease the pro-inflammatory response of BV2 microglia to lipopolysaccharide [70]. However, in the previous study, the BV2 were immune-activated for four hours, and we activated the BV2 for 16 h, possibly indicating a time-dependent effect. Also, CBD, but not NTI-164, reduced the release of IL-2 from immune-activated BV2 microglia. CBD has previously been shown to decrease IL-2 secretion by lymphocytes [101]. There is evidence for altered IL-2 signalling in people with ASD [102], but its role as a potential peripheral biomarker was not demonstrated in a recent meta-analysis [103]. NTI-164 or CBD also did not affect IL-5, IL-4, or IL-10 levels, which contrasts with previous studies showing changes in the production of these cytokines induced by CBD under inflammatory conditions [104]. The variability in our findings and previous studies could be due to differences in the composition of *Cannabis* extracts from different strains, which is why detailed analysis of extract composition is critical. We also cannot rule out dose-dependent effects [105], which needs to be explored in future studies. The neuroprotective and anti-inflammatory profiles of NTI-164 will be further characterised using in vivo models of neuroinflammation and ASD-like phenotypes.

The underlying positive mechanisms of phytocannabinoids are challenging to characterise due to the complexity and diversity of the pharmacodynamic profile. However, several mechanisms for attenuation of inflammation have been proposed that might explain the potential for cannabinoids found in NTI-164 to decrease cytokines and COX2 expression. A recent study showed that the anti-inflammatory effect of CBD involved inhibiting the NF-B-dependent signalling pathway activated by oxidative-stress-activated NF-κB-dependent signalling pathway through regulation of NADPH production and glucose uptake [106]. In addition to the potential modulation of intracellular antioxidant pathways, other pharmacological anti-inflammatory mechanisms include modulating calcium signalling [67] and through increased peroxisome proliferator-activated receptor γ-dependent activation [107].

In addition to its anti-inflammatory effect, NTI-164 treatment significantly increased the proliferation of neural progenitors and promoted the survival of neurons in an excitotoxic paradigm, while CBD did not. This could indicate the observed effect was due to botanical synergy or due to the CBD dose response. However, Luján and Valverde suggest that CBD exerts pro-neurogenic effects on newborn neurons but not progenitors [108]. However, the effect of phytocannabinoid combinations such as NTI-164 on the proliferation of neural progenitor cells requires further investigation. Several biological mechanisms involved in CBD-mediated neuroplasticity, protection and survival in adult neurons have been described [109], including extracellular-signal-regulated kinases [110], glycogen synthase kinase 3β signalling [58,111], and mammalian target of rapamycin pathways [112]. Given the interaction between phytocannabinoids and ECS, the molecular mechanisms underlying the synergistic effect of *Cannabis* constituents on neurogenesis in preclinical models need further investigation.

## 5. Conclusions

We explored the anti-inflammatory and neuroprotective effects of the chemically characterised full-spectrum *Cannabis* extract NTI-164 in immune-activated cells. NTI-164 attenuated inflammatory cytokines release and promoted neuronal survival; isolated CBD did not. Together, these findings suggest—but cannot establish—that the anti-inflammatory effect of NTI-164 is likely due to the synergistic interaction of the *Cannabis* extract derivatives rather than CBD alone. The entourage effect of full-spectrum *Cannabis* derivatives must be confirmed in vivo, especially with studies designed to assess the brain penetrance of the psychoactive compounds in the extract. Before any in vivo efficacy studies, it will also be imperative to undertake detailed pharmacokinetic/pharmacodynamic studies to inform the choice of treatment dose and timing schedules. Future studies should characterise the effects of NTI-164 on key anti-inflammatory and immunomodulatory mechanisms involved in the pathogenesis and progression of neurological diseases in vivo. Furthermore, the synergistic effect of phytocannabinoids in modulation of microglial cell functions warrants further investigation with combinations of cannabinoid components. Further examination of the optimal dose of NTI-164 and duration of treatment may also improve efficacy and expected outcomes.

## Figures and Tables

**Table 1:** Primary and secondary antibodies.

Antibody	Concentration	Supplier
Mouse α-ARG1	1:250	Abcam, Singapore. Cat# AB239731
Rabbit α-COX2	1:500	Abcam, Singapore. Cat# AB15191
Rabbit α-iNOS	1:250	Abcam, Singapore. Cat# AB178945
Mouse α-Beta III Tubulin	1:500	R&D Systems Minneapolis, MN, USA., Cat# MAB1195
Goat α-Rabbit IgG, Alexa Fluor 555	1:500	Invitrogen, Waltham, MA, USA. Cat# A-21428
Goat α-Mouse IgG, Alexa Fluor 488	1:500	Invitrogen, Waltham, MA, USA. Cat# A-11001

Abbreviations. α-: anti; ARG1: Arginase-1; COX2: cyclooxygenase-2; IgG: Immunoglobulin G; iNOS: inducible nitric oxide synthase.

## Data Availability

All raw data will be made available on request. A preprint has previously been published; doi: https://doi.org/10.1101/2024.01.10.575133 (accessed on 4 October 2024).

## Supplementary Materials

The following supporting information can be downloaded at: https://www.mdpi.com/article/10.3390/biom14111434/s1, Figure S1: Description of, (A) changes in inflammatory antigens after inflammation exposure and injury severity in the glutamate exposure paradigm for (B) mitochondrial output, and (C) cell morphology.
