# Supplementary material for: Evaluation of the Efficacy of a Full-Spectrum Low-THC Cannabis Plant Extract Using In Vitro Models of Inflammation and Excitotoxicity
Source: Biomolecules. 2024 Nov 11;14(11):1434. doi: 10.3390/biom14111434 (PMC11592195; doi:10.3390/biom14111434)
Supplement: Supplementary file 1 [file biomolecules-14-01434-s001.zip › biomolecules-3174897-supplementary.pdf]

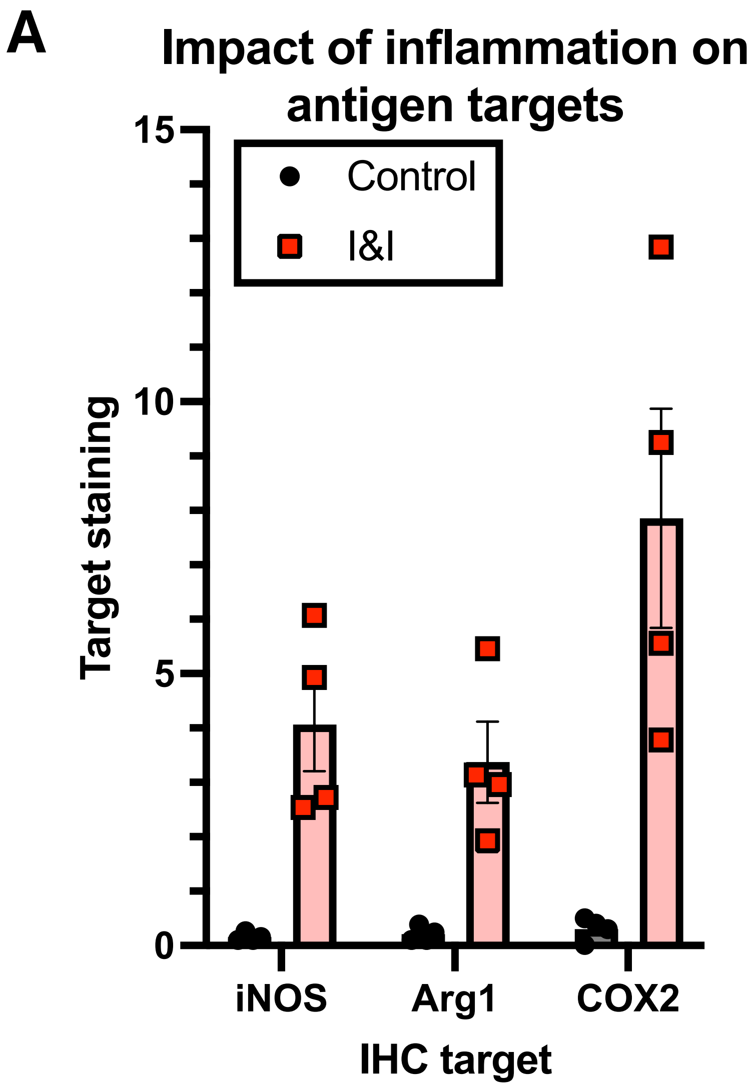

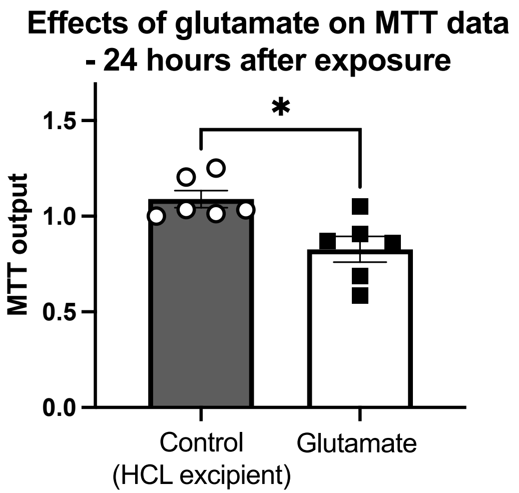

Paired t test<br>Tabular results	
Table Analyzed	Glutamate injury only
Column B	Glutamate only
vs.	vs.
Column A	Control (HCL excipient)
**Paired t test**	
P value	0.0172
P value summary	*
Significantly different (P < 0.05)?	Yes
One- or two-tailed P value?	Two-tailed
t, df	t=3.506, df=5
Number of pairs	6
**How big is the difference?**	
Mean of differences (B - A)	-0.2625
SD of differences	0.1834
SEM of differences	0.07487
95% confidence interval	-0.4549 to -0.07005
R squared (partial eta squared)	0.7109
**How effective was the pairing?**	
Correlation coefficient (r)	0.1607
P value (one tailed)	0.3805
P value summary	ns
Was the pairing significantly effective?	No

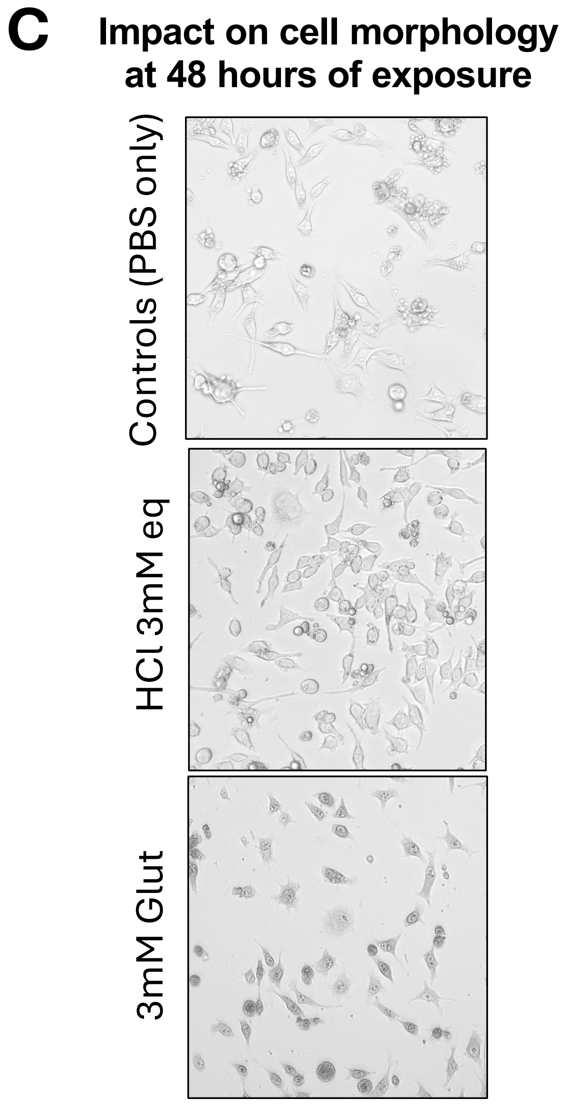

Supplemental Figure 1. Description of, (A) changes in inflammatory antigens after inflammation exposure and injury severity in the glutamate exposure paradigm for (B) mitochondrial output, and (C) cell morphology.
